# Hints and Suggestions as Aids in the Care and Preservation of the Teeth

**Published:** 1896-04

**Authors:** 


HINTS AND SUGGESTIONS AS AIDS IN THE CARE AND PRESERVATION OF THE TEETH AND THE RELATION OF THE DENTAL ORGANS TO OUR HEALTH. By Charles G. Pease, M. D., D. D. S., Professor of Oral Surgery in the Metropolitan Post-Graduate School of Medicine. Published for the benefit of the Laity. New York: Bœricke, Runyon & Ernesty. 1895. 46 pages. Price, $1.00, net.

This admirable little book deserves a place in every household. It teaches the care of the teeth by teaching the whole theory of the diseases of the teeth and the principles upon which the dentist treats these dental diseases.

The book starts out with an explanation of the theory of caries. This theory is that it is due to the action of an acid which is the product of micro-organisms that generate fermentation. The acid dissolves out the lime-salts at the point of contact, exposing the organic matter, which in its turn is acted on by the ferment, and so the phenomena are continued. This view is backed up by practical laboratory experiments such as that of Dr. Margitot, of Paris, who placed sound teeth into a solution of sugar in water, and allowed the solution to undergo fermentation. At the end of two years the solution was found to be markedly acid, and the teeth completely decalcified.

There are short chapters on disintegration of teeth occurring in early motherhood on local and systemic conditions favoring decay, upon the use of the tooth-brush, of floss silk, of the tooth-pick and of dentifrices. Hygiene, physical exercise, and food receive attention, and the proper mastication of food enjoined. The habit of gum chewing is denounced as pernicious, and a vigorous protest against its encouragement is entered.

The proper occlusion of the teeth is dwelt upon, and the preservation of the temporary teeth.

Face-ache, alveolar abscess and tartar upon the teeth; pyorrhœa alveolaris, recession of the gums, sensitive dentine, and erosion of the teeth are explained, and the correction of irregularity. Other subjects treated are: facial orthopedia, gold crowns, extraction of teeth, artificial teeth, etc. All these chapters are short and plainly written to reach the popular mind. There are no complicated scientific discussions, and so the book is well suited for the purpose for which it was designed. There are also a few indications for the use of homœopathic remedies, which are, however, mixed up with one or two formulæ for mouth washes in cases of aphthæ and ulcers, and for soreness about roots on pressing teeth.

The book is a convenient one for a doctor to hand to his patient whom he finds insufficiently careful of those pearls of great price, a double row of fine healthy teeth.

